# Genome Sequences of Three *Lactobacillus* Species Strains of the Stomach of the White-Footed Deermouse (Peromyscus leucopus)

**DOI:** 10.1128/MRA.00963-19

**Published:** 2019-10-03

**Authors:** Khalil Bassam, Ana Milovic, Alan G. Barbour

**Affiliations:** a Department of Microbiology and Molecular Genetics, University of California Irvine, Irvine, California, USA; b Department of Medicine, University of California Irvine, Irvine, California, USA; University of Maryland School of Medicine

## Abstract

Three colony types of *Lactobacillus* were isolated from the stomach of LL colony stock Peromyscus leucopus deermice, a reservoir for several human zoonoses. Genome sequences revealed two isolates to be new strains of Lactobacillus animalis and Lactobacillus reuteri. The third was distinct from known species and was provisionally designated *Lactobacillus* sp. strain LL6.

## ANNOUNCEMENT

Peromyscus leucopus, a widely distributed, abundant mammal in North America, is an important reservoir for agents of tick-borne zoonoses, including Lyme disease, babesiosis, anaplasmosis, and a viral encephalitis (1). The genus *Peromyscus* clusters with hamsters and voles rather than with murids such as Mus musculus (2). *P. leucopus* is the target for field-delivered oral vaccines (3), but little is known about the gastrointestinal (GI) microbiota of this or other *Peromyscus* species.

During study of the GI tract of animals of *P. leucopus* LL stock (Peromyscus Genetic Stock Center, University of South Carolina), we identified three types of *Lactobacillus* in the stomachs, ceca, and feces of both females and males. The organisms were isolated from stomach contents on Rogosa SL agar (Sigma-Aldrich) plates in a candle jar at 37°C and were distinguished by colony morphologies. Single colonies of each were cultivated in Difco MRS broth (Becton, Dickinson). High-molecular-weight genomic DNA was extracted from harvested cells with a Qiagen Genomic-tip 100/G kit.

Their genomes were sequenced by two methods, (i) for long reads on an Oxford Nanopore Technology (ONT) MinION Mk1B instrument with a ligation sequencing kit, R9.4.1 flow cell, MinKNOW v. 19.6.8 for primary data acquisition, and Guppy v. 3.2.4 for base calling with default settings and (ii) for short reads on an Illumina MiSeq instrument with paired-end v2 microchemistry and 150 cycles. The library was constructed using the NEXTflex rapid DNA-seq kit (Bioo Scientific), the quality of sequencing reads was analyzed using FastQC (Babraham Bioinformatics), and reads were trimmed of Phred scores of <15 and corrected for poor-quality bases using Trimmomatic (4).

A hybrid assembly of each genome was carried out with Unicycler v. 0.4.7 (5) with default settings and 16 threads on the high-performance computing cluster of the University of California Irvine. Complete, circularly permuted chromosomes were obtained for two of the isolates (LL1 and LL7), and two contigs of 1.75 Mb and 0.32 Mb, respectively, were obtained for the third (LL6) (Table 1). Annotation was provided by the NCBI Prokaryotic Genome Annotation Pipeline (6). By the criterion of 16S ribosomal sequence identity of ≥99%, isolates LL1 and LL7 were identified as Lactobacillus animalis and Lactobacillus reuteri, respectively. Other sequences of these organisms confirmed those classifications.

**Table 1:** Resources from this study, sequencing statistics, and characteristics of chromosomes and plasmids of three *Lactobacillus* spp.

Species	Strain	BioProject accession no.	BioSample accession no.	SRA accession no. (Illumina, ONT)	No. of ONT reads (×10^6^)/*N*_50_ (kb)	Mean coverage (×)		Size (bp)		G+C content (%)		GenBank accession no.	
						ONT	Illumina	Chromosome	Plasmid	Chromosome	Plasmid	Chromosome(s)	Plasmid
Lactobacillus animalis	LL1	PRJNA533837	SAMN11468794	SRX5831611, SRX5831612	2.31/6.11	2,098	182	2,280,577	4,016	41.0	37.1	CP039849	MK858222
Lactobacillus reuteri	LL7	PRJNA554696	SAMN12280509	SRX6494060, SRX6494061	1.52/0.87	423	218	2,205,740	178,977	38.9	38.1	CP041676	CP041677
*Lactobacillus* sp.	LL6	PRJNA554698	SAMN12280527	SRX6492358, SRX6492357	2.73/6.21	2,601	278	2,067,236	6,969	33.5	36.4	VLLR01000001 (contig 1), VLLR01000002 (contig 1)	VLLR01000003

L. reuteri LL7 had another replicon (Table 1) that had coding sequences for a Rep_3 (pfam01051) plasmid replication initiation protein and multiple transposases. This is consistent with a type of megaplasmid observed in other lactobacilli (7).

Isolate LL6 could be not be identified with a known *Lactobacillus* species in the genome database. Over its full-length 16S rRNA gene sequence, the closest organism was Lactobacillus amylolyticus at 95.6% nucleotide identity. The identities of LL6’s coding sequences for the core genome genes *recA*, *rpoB*, *gyrA*, and *rplB* were no more than 83%, 87%, 81%, and 94%, respectively, with any other *Lactobacillus* sp. These distances suggest that this represents a previously unknown taxon, provisionally designated *Lactobacillus* sp. strain LL6.

### Data availability.

The strains described here are available from the corresponding author. Table 1 provides the publicly available resources from this study with hyperlinks to repositories.
